# Degradation of Polymer Materials in the Environment and Its Impact on the Health of Experimental Animals: A Review

**DOI:** 10.3390/polym16192807

**Published:** 2024-10-03

**Authors:** Xiyu Zhang, Zhenxing Yin, Songbai Xiang, Huayu Yan, Hailing Tian

**Affiliations:** 1 Department of Chemistry, National Demonstration Centre for Experimental Chemistry Education, Yanbian University, Yanji 133002, China; 18943306230@163.com (X.Z.); yinzx@ybu.edu.cn (Z.Y.); 17671374725@163.com (S.X.); 2 Laboratory Animal Center, Yanbian University, Yanji 133002, China

**Keywords:** polymeric degradation, environmental pollution, experimental animal, toxicity

## Abstract

The extensive use of polymeric materials has resulted in significant environmental pollution, prompting the need for a deeper understanding of their degradation processes and impacts. This review provides a comprehensive analysis of the degradation of polymeric materials in the environment and their impact on the health of experimental animals. It identifies common polymers, delineates their degradation pathways, and describes the resulting products under different environmental conditions. The review covers physical, chemical, and biological degradation mechanisms, highlighting the complex interplay of factors influencing these processes. Furthermore, it examines the health implications of degradation products, using experimental animals as proxies for assessing potential risks to human health. By synthesizing current research, the review focuses on studies related to small organisms (primarily rodents and invertebrates, supplemented by fish and mollusks) to explore the effects of polymer materials on living organisms and underscores the urgency of developing and implementing effective polymer waste management strategies. These strategies are crucial for mitigating the adverse environmental and health impacts of polymer degradation, thus promoting a more sustainable interaction between human activities and the natural environment.

## 1. Introduction

Polymeric materials, particularly synthetic polymers, have become integral to various fields in modern society due to their superior physical and chemical properties. Their widespread applications span packaging [1], construction [2], electronics [3], and healthcare [4]. The convenience and versatility offered by these materials have led to an exponential increase in their production and usage. However, this surge in consumption has brought significant environmental concerns. Owing to their inherent stability and resistance to degradation, polymeric materials persist in the environment, leading to substantial pollution issues [5,6].

The degradation process of polymeric materials in the environment is multifaceted, involving physical, chemical, and biological mechanisms. Physical degradation typically involves mechanical processes such as abrasion and fragmentation, while chemical degradation includes processes like photo-oxidation, thermal degradation, and hydrolysis [7,8]. Biological degradation involves the breakdown of materials by microorganisms, such as bacteria and fungi [9]. The choice of degradation mechanism is influenced by the higher-order structure of the polymer material, including crystallinity, crosslinking degree, and macroscopic arrangement [10]. The types and properties of degradation products vary widely depending on the material type and environmental conditions, such as temperature, light exposure, and the presence of microorganisms [11]. These degradation products, often microscopic in size, can disperse widely and persist in various environmental matrices, posing threats to ecosystems and potentially entering the food chain [12].

The persistence and accumulation of polymeric materials in the environment raise significant ecological and health concerns. These materials, particularly microplastics, have been detected in oceans, rivers, soils, and even the air [13,14,15]. They can adsorb and concentrate toxic chemicals from the environment, acting as vectors for pollutants [16]. The ingestion of these materials by wildlife, including fish, birds, and terrestrial animals, can lead to physical blockages, toxicological effects, and transfer up the food chain, ultimately impacting human health [17,18]. The exact mechanisms and extent of these impacts remain a critical area of research.

As well known, microplastics and other polymer degradation products are ingested by a wide range of organisms, from zooplankton to large mammals. This ingestion can cause physical harm, such as gastrointestinal blockages, and can introduce toxic chemicals into the organism’s system, leading to various health effects [19]. For instance, microplastics have been found to cause oxidative stress [20], inflammation [21], and even genotoxic effects [22] in some animals. Furthermore, the potential for these materials to bioaccumulate and biomagnify through food webs poses a significant risk to higher trophic levels, including humans.

Despite the growing body of research focusing on the environmental degradation of polymeric materials, systematic studies examining the health effects of these degradation products on experimental animals are relatively scarce. Experimental animals, such as rodents and aquatic organisms, are essential models in environmental toxicology research [23,24]. They provide valuable insights into the potential health risks posed by environmental pollutants, including polymer degradation products. Understanding these effects is crucial for assessing the biosafety of these materials and for developing effective regulatory and mitigation strategies [25]. Experimental studies on animals have begun to shed light on the various health impacts of polymer degradation products. Noteworthy, is tha, exposure to microplastics can lead to a range of adverse health effects in rodents, including alterations in gut microbiota, liver damage, and reproductive toxicity [26,27]. Similarly, aquatic organisms exposed to degraded polymers have shown signs of stress, developmental abnormalities, and increased mortality rates [28]. These findings highlight the urgent need for more comprehensive studies to fully understand the long-term health implications of polymer degradation products.

This review aims to provide a comprehensive overview of the common types of polymers, the pathways through which they degrade in various environmental conditions, and the specific degradation products that result. Additionally, it was explored how these degradation products affect the health of experimental animals, which can serve as indicators for potential human health risks. The health effects of these degradation products on experimental animals, including acute and chronic toxicity, reproductive toxicity, immunotoxicity and carcinogenicity, were also discussed. By synthesizing current research findings, it seeks to highlight the importance of developing effective strategies to manage polymer waste and mitigate its adverse effects on the environment and public health. It is hoped that this review will serve as a valuable resource for researchers, policymakers, and industry stakeholders, promoting the sustainable development of polymeric materials and contributing to the protection of environmental and public health.

## 2. Common Polymeric Materials and Their Degradation Products

Polymeric materials play crucial roles in modern industries and daily life due to their versatility and durability. However, their widespread use has led to concerns regarding their environmental impact, particularly their persistence and the generation of potentially harmful degradation products upon exposure to environmental conditions [29]. This section provides an in-depth exploration of common polymeric materials, their typical degradation pathways, and the properties of the resulting degradation products.

### 2.1. Common Polymeric Materials

Polyethylene (PE), a widely used thermoplastic, is highly valued for its excellent chemical resistance and low cost. It is often used in the form of high-density polyethylene (HDPE) and low-density polyethylene (LDPE) for the production of plastic films and containers [30]. Ultra-high molecular weight polyethylene (UHMWPE) fibers with extremely high strength and lightweight properties can be used to prepare bulletproof vests. Each form of PE has different properties that affect their environmental fate. PE primarily degrades through physical and oxidative mechanisms. Physical degradation involves fragmentation into smaller pieces due to mechanical stresses like abrasion and weathering [31]. Oxidative degradation occurs when PE is exposed to UV radiation, which initiates photochemical reactions leading to the formation of free radicals and subsequent chain scission [32,33]. The degradation of PE results in the formation of smaller hydrocarbons such as alkanes, alkenes, ketones, alcohols, and carboxylic acids [34]. These products contribute to the persistence of microplastics in the environment, which can be ingested by marine organisms and enter the food chain.

Polypropylene (PP) is another widely used thermoplastic known for its robustness, chemical resistance, and versatility in applications ranging from packaging to automotive components [35]. Moreover, the biocompatibility and electrical insulation properties of polypropylene make it suitable for the medical and electronic fields. Similar to PE, PP undergoes physical and oxidative degradation. Physical degradation involves mechanical wear and tear, while oxidative degradation occurs through the action of UV radiation and thermal exposure, leading to chain scission and the formation of carbonyl groups [36]. Upon degradation, PP releases hydrocarbons, ketones, aldehydes, and carboxylic acids [37]. These degradation products contribute to environmental pollution, particularly in aquatic ecosystems where they can adsorb persistent organic pollutants and disrupt marine life.

Polyvinyl chloride (PVC) is widely used in construction, healthcare, and consumer goods due to its durability and fire-resistant properties [38]. However, PVC presents unique challenges due to the release of toxic compounds during degradation. PVC degrades primarily through photo-oxidation and thermal degradation. UV radiation initiates photochemical reactions that lead to dehydrochlorination, releasing hydrogen chloride (HCl) and forming conjugated polyenes. Thermal degradation also contributes to the release of HCl and the formation of aromatic and chlorinated hydrocarbons [39]. The degradation of PVC releases toxic chemicals such as HCl, chlorinated hydrocarbons, and dioxins [40]. These compounds pose significant risks to human health and the environment, particularly in terms of bioaccumulation and toxicity in aquatic and terrestrial ecosystems.

Polystyrene (PS) is widely used in disposable packaging, insulation, and food containers due to its low cost and insulation properties [41]. However, PS is highly resistant to degradation, contributing to its persistence in the environment. PS degrades primarily through photo-oxidative and thermal processes. UV radiation breaks down the polymer chains, leading to the formation of styrene monomers, oligomers, and aromatic hydrocarbons. Thermal degradation at high temperatures also contributes to the release of volatile organic compounds (VOCs). The degradation products of PS include styrene monomers, benzaldehyde, and other aromatic compounds [42,43,44]. These chemicals can leach into the environment, posing risks to aquatic organisms and potentially entering the food chain through bioaccumulation.

Polyethylene terephthalate (PET) is widely used in beverage bottles, textiles, and packaging due to its strength, clarity, and recyclability [45]. However, PET’s resistance to degradation poses challenges for environmental sustainability. PET degrades primarily through hydrolysis and photo-oxidation. Hydrolytic degradation occurs in the presence of water, leading to the cleavage of ester bonds and the formation of terephthalic acid and ethylene glycol. UV radiation accelerates photo-oxidative degradation, generating carbonyl compounds and other oxidized products. The degradation products of PET include terephthalic acid, ethylene glycol, and various carbonyl compounds [46,47,48]. These products can persist in the environment, contributing to microplastic pollution and posing ingestion risks to marine organisms.

Polyurethane (PU) is used in foams, coatings, and adhesives due to its versatility and durability. In addition, it is widely used in the manufacturing of coatings, adhesives, and sealants. However, PU’s complex structure makes it challenging to degrade, requiring specialized processes or microbial activity. PU degrades through hydrolysis, oxidative degradation, and microbial action. Hydrolysis breaks down the urethane and ester bonds, while oxidative degradation involves the formation of carbonyl groups under UV radiation [49]. Microorganisms produce enzymes that can hydrolyze PU, leading to the formation of biodegradable metabolites. Upon degradation, PU releases polyols, amines, and other breakdown products. Microbial degradation can convert PU into carbon dioxide, water, and biomass, depending on the microbial community and environmental conditions [50,51,52].

Polycarbonate (PC) is used in optical media, eyewear, and durable goods due to its clarity, impact resistance, and high-temperature stability. PC is also used in the production of transparent insulation panels for automotive parts, electronic device casings, and building materials. However, PC’s susceptibility to degradation and the release of harmful compounds pose environmental concerns. PC degrades through the hydrolysis of the carbonate linkages, especially under acidic or alkaline conditions [53]. UV radiation also induces photo-oxidative degradation, leading to chain scission and the formation of phenolic compounds. The degradation of PC produces bisphenol A (BPA), a compound known for its endocrine-disrupting properties [54]. Other degradation products include phenolic compounds and aromatic hydrocarbons [55], which can persist in the environment and pose risks to ecosystems and human health.

Polylactic acid (PLA) is a biodegradable plastic widely used in various applications, including packaging, textiles, medical devices, etc. Usually, high temperature and humidity conditions can cause the ester bonds of PLA to break, generating lactic acid monomers, which are further degraded into carbon dioxide and water [56]. Biodegradation is the main degradation method of PLA. In a suitable industrial composting environment, microorganisms degrade PLA into carbon dioxide and water through esterification enzyme action [57]. This process is usually carried out in a composting environment with higher temperature and humidity, and the degradation rate is faster.

Polybutylene succinate (PBS) is a biodegradable plastic that has attracted attention for its excellent biocompatibility and biodegradability. The physical degradation of PBS is mainly caused by ultraviolet irradiation and mechanical wear, resulting in PBS chain breakage and the formation of smaller particles. Chemical degradation occurs through hydrolysis reactions, especially in humid environments, where the ester bonds of PBS break, releasing succinic acid and terephthalic acid. Biodegradation is the main pathway for the degradation of PBS. Under suitable environmental conditions, microorganisms and fungi will use esterases to degrade PBS into simpler organic compounds. Degradation products include succinic acid and terephthalic acid, which typically have low environmental toxicity and minimal impact on ecosystems [58]. However, high concentrations of degradation products may cause certain toxicity to certain organisms, therefore a further evaluation of the long-term impact of PBS degradation products on the environment and ecosystem is needed.

Poly (β-hydroxybutyrate) (PHB) is a natural biodegradable polymer synthesized by microorganisms and can be used as an energy storage substance in living organisms. In a humid environment, PHB undergoes a hydrolysis reaction, and its ester bonds break to form β-hydroxybutyric acid (HB), which accelerates under higher humidity and temperature conditions [59]. Biodegradation is the main degradation mode of PHB, and microorganisms degrade PHB into HB through esterases, which are then further metabolized into carbon dioxide and water [58]. The degradation product HB has biodegradability and is relatively environmentally friendly, but it may have an impact on certain microbial communities during PHB degradation.

While polymeric materials offer valuable properties for diverse applications, their persistence and the generation of potentially harmful degradation products present significant environmental challenges. Addressing these challenges requires a comprehensive understanding of degradation mechanisms and the development of sustainable practices for polymer use, recycling, and waste management.

### 2.2. Persistence of Polymeric Degradation Products

**Figure 1:**
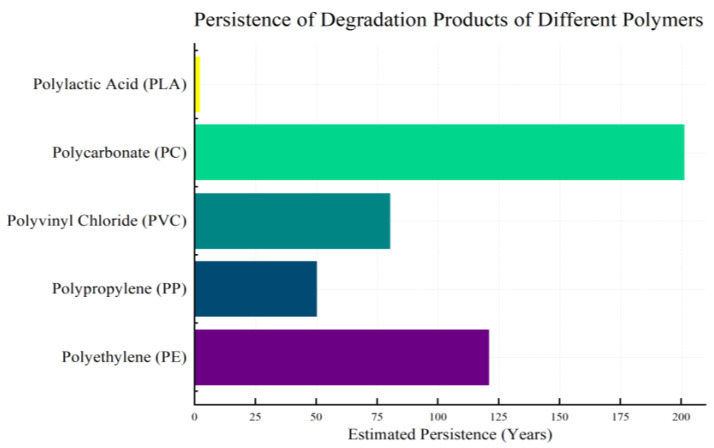
Persistence of the degradation products from polyethylene, polypropylene, polyvinyl chloride, polycarbonate, and polylactic acid.

Polymeric materials degrade into various products that often exhibit persistence in the environment. The duration of persistence for polymer degradation products differs based on various factors, including their chemical structure, environmental conditions, and method of degradation. Some degradation products may persist in the environment for a brief few years, while others may remain for decades or longer. Figure 1 displays the environmental persistence (in years) of the degradation products from five common polymers (polyethylene, polypropylene, polyvinyl chloride, polycarbonate, and polylactic acid). The figure reveals that degradation products from polycarbonate (PC) and polyethylene (PE) have a longer persistence, whereas those from polylactic acid (PLA) are more short-lived. This duration of persistence is determined by their ability to be effectively degraded by microorganisms or other environmental factors.

Microplastics, for example, are small plastic particles (<5 mm) that result from the fragmentation of larger plastic items or are manufactured at small sizes for specific uses [60]. These microplastics can persist in marine and terrestrial environments for extended periods due to their resistance to biological, chemical, and physical degradation processes. Their small size and buoyancy enable them to disperse widely across the globe through ocean currents and atmospheric transport, contaminating remote and pristine environments far from their sources [61]. Chemical breakdown products from polymers, such as plasticizers, flame retardants, and monomers [62], can also exhibit persistence depending on their chemical stability and resistance to biodegradation. Some of these compounds can bioaccumulate in organisms through the food chain, potentially reaching concentrations that pose risks to higher trophic levels, including humans [63].

### 2.3. Transformation of Polymeric Degradation Products

In the environment, degradation products undergo various transformation processes that can alter their chemical properties and environmental fate. Microplastics, subjected to physical weathering processes such as UV radiation, abrasion, and microbial degradation, break down into smaller particles known as nanoplastics [60]. Nanoplastics may exhibit different behaviors and interactions with organisms compared to larger microplastics, potentially affecting their toxicity and environmental impact. In addition, chemical breakdown products can undergo biotic and abiotic transformations that modify their chemical structures and toxicity profiles [64]. Microorganisms play a crucial role in biotransforming organic pollutants, breaking them down into less harmful substances or metabolizing them into more toxic intermediates. Environmental factors such as temperature, pH, and the presence of specific microbial communities influence the rates and pathways of these transformation processes [65].

## 3. Mechanisms of Polymeric Material Degradation

The degradation of polymeric materials is a complex and multifaceted process involving several mechanisms, including physical, chemical, and biological pathways. These processes can occur independently or synergistically, resulting in changes at both the macroscopic and microscopic levels [66]. Understanding these degradation mechanisms is crucial for assessing the behavior and potential environmental impacts of polymeric materials.

### 3.1. Physical Degradation

Physical degradation primarily refers to the breakdown of polymeric materials through mechanical forces. Common forms of physical degradation include abrasion, tearing, and fragmentation. These processes are typically induced by external physical stresses such as weathering, friction, and impact [31,67]. Environmental factors such as temperature fluctuations and ultraviolet (UV) radiation can also accelerate physical degradation. For example, in high-temperature environments, the thermal expansion and contraction of materials can lead to the formation and propagation of cracks, while UV radiation can cause surface embrittlement, making the materials more prone to fracture [68].

Abrasion occurs when polymer surfaces are worn down by friction or rubbing against other materials or particles. This is particularly common in environments with significant particulate matter, such as sandy or dusty areas. Over time, abrasion can lead to the gradual thinning and weakening of the polymeric material. Fragmentation involves the breaking of polymers into smaller pieces due to mechanical stresses such as impacts, bending, or stretching [69,70]. This process can be accelerated by environmental factors like temperature fluctuations, which cause expansion and contraction cycles that induce stress fractures. Cracking is often a result of both mechanical and environmental stresses. For example, repeated exposure to UV radiation can cause surface embrittlement, leading to the formation of microcracks. These cracks can propagate under mechanical stress, eventually leading to the fragmentation of the material [71,72].

### 3.2. Chemical Degradation

Chemical degradation involves the breaking of chemical bonds within the polymer chains, leading to changes in the material’s chemical structure and properties [73]. This degradation can occur through several mechanisms, including photochemical degradation, thermo-oxidative degradation, and hydrolytic degradation.

#### 3.2.1. Photochemical Degradation

**Figure 2:**
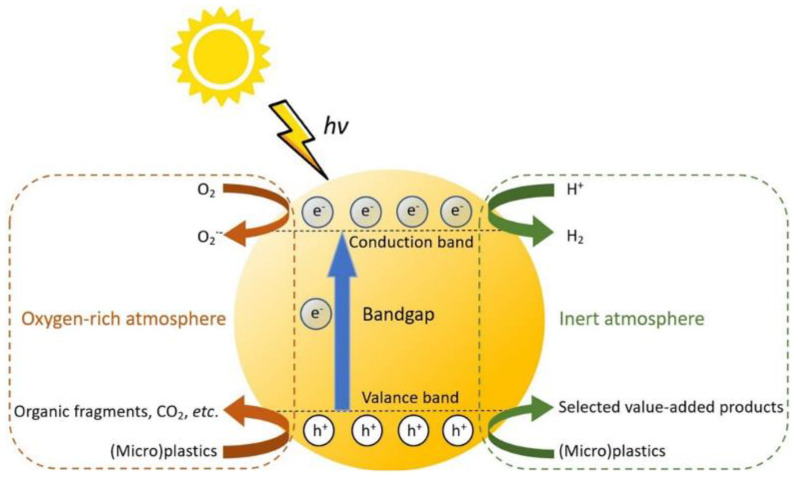
Schematic diagram of photocatalytic reaction mechanism in different environments, reprinted with permission from [76], 2023, Royal Society of Chemistry.

Exposure to UV and visible light can initiate the direct absorption of photons by high polymer macromolecules to produce excited states, resulting in the cleavage of polymer chains [74]. When the photocatalytic reaction is in an oxygen-rich state, oxygen facilitates the formation of reactive oxygen species (ROS) such as peroxides and free radicals, which further react with the polymer, causing chain scission and cross-linking that degrade the material [75]. For example, polyethylene and polypropylene are particularly susceptible to photo-oxidative degradation due to their chemical structure. Another type of degradation is performed in an inert atmosphere; the hole-generated photons drive the transformation of (micro)plastic to produce relatively highly selective value-added chemicals and H_2_ [76]. Figure 2 illustrates their mechanism.

#### 3.2.2. Thermo-Oxidative Degradation

Elevated temperatures can cause the thermal decomposition of polymers, a process known as thermo-oxidative degradation [77]. This degradation is often accelerated by the presence of oxygen, leading to the formation of oxidative degradation products such as ketones, alcohols, and acids [78]. Thermo-oxidative degradation can significantly alter the mechanical properties of polymers, making them brittle and less durable. For instance, polyvinyl chloride (PVC) can undergo dehydrochlorination at high temperatures, resulting in the formation of conjugated polyenes and subsequent embrittlement [79].

#### 3.2.3. Hydrolytic Degradation

Some polymers, like polyesters and polyamides, can undergo hydrolysis in the presence of water, leading to the cleavage of ester or amide bonds [80,81]. This process generates lower molecular weight products such as acids and alcohols. Hydrolytic degradation is particularly significant in humid environments, where moisture can penetrate the polymer matrix and catalyze the hydrolysis reaction [82]. An example is the hydrolytic degradation of polyethylene terephthalate (PET) in aquatic environments, which leads to the formation of terephthalic acid and ethylene glycol [46].

Overall, understanding these chemical degradation mechanisms is crucial for predicting the longevity and performance of polymeric materials under various environmental conditions and for developing strategies to enhance their stability.

### 3.3. Biological Degradation

Biological degradation refers to the breakdown of polymeric materials by microorganisms, such as bacteria, fungi, and algae [83]. This process can be divided into two main stages. One stage is primary biodegradation. Microorganisms secrete extracellular enzymes that act on the polymer surface, breaking down the long polymer chains into smaller fragments. These enzymes, including proteases, lipases, and cellulases, target specific chemical bonds within the polymer. For example, the enzyme cutinase can hydrolyze the ester bonds in PET, leading to its degradation into terephthalic acid and ethylene glycol [84,85,86]. The other one is secondary biodegradation. The smaller molecular fragments produced during primary biodegradation are further metabolized by microorganisms. These fragments are absorbed and utilized as carbon and energy sources, eventually being broken down into simple inorganic compounds such as carbon dioxide, water, and biomass [87]. The efficiency and rate of biodegradation depend on factors such as the chemical structure of the polymer, its molecular weight, its crystallinity, and the environmental conditions (e.g., temperature, pH, and the presence of specific microorganisms) [88].

Biodegradation is influenced by the intrinsic properties of the polymer as well as the environmental conditions. Polymers with readily hydrolyzable bonds, such as polyesters and polyurethanes, tend to degrade more easily in biological environments. Additionally, factors like temperature, moisture, and the presence of specific microbial communities play crucial roles in determining the rate and extent of biodegradation [89].

### 3.4. Environmental Factors Affecting Degradation

**Figure 3:**
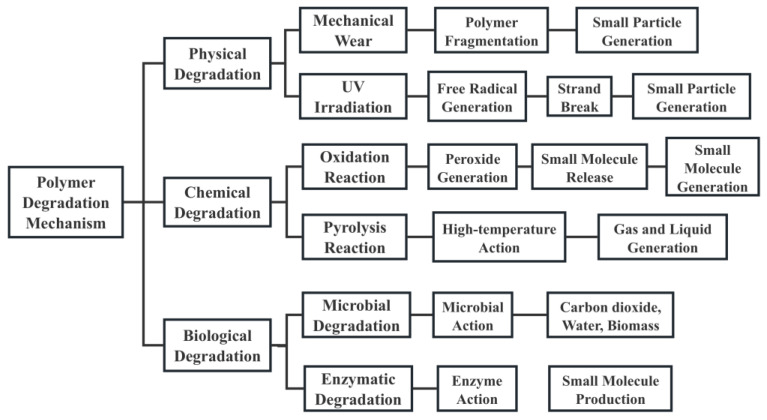
Process diagram of polymer degradation mechanism.

The degradation mechanism of polymers is influenced by a variety of environmental factors, including physical, chemical, and biological processes [90,91]. UV irradiation, temperature fluctuations, and the participation of microorganisms can significantly impact the rate and product form of the degradation process (as illustrated in Figure 3). Among these influencing factors, temperature is a primary factor, as higher temperatures generally enhance the rates of chemical and biological degradation processes. This enhancement occurs because increased temperatures elevate the kinetic energy of molecules, thereby facilitating bond breakage and enzymatic activities. For example, in warmer climates, polymers might degrade faster due to heightened molecular motion and accelerated biochemical reactions. Conversely, in colder environments, degradation processes are slowed down due to reduced molecular mobility and diminished microbial activity, resulting in prolonged polymer lifespan [92]. Humidity also plays a crucial role, particularly in hydrolytic and biological degradation. The presence of water is essential for hydrolysis reactions and provides a conducive medium for microbial growth and enzyme activity. High humidity conditions promote the absorption of moisture into the polymer matrix, accelerating hydrolytic degradation. For instance, in tropical regions with high humidity, polymers are more prone to rapid degradation. On the other hand, in arid environments, the scarcity of moisture limits hydrolytic and biological degradation, thus preserving the integrity of polymers for a longer duration [93]. Light exposure, especially to UV and visible light, is another critical factor influencing polymer degradation. UV radiation can significantly accelerate photo-oxidative degradation by providing the energy required to break chemical bonds and generate reactive intermediates. In regions with high UV radiation, such as tropical and subtropical areas, polymers are more susceptible to rapid degradation. However, the incorporation of UV stabilizers and antioxidants in polymer formulations can mitigate this effect by absorbing or neutralizing harmful UV radiation, thereby extending the material’s lifespan. Lastly, oxygen availability is crucial for oxidative degradation processes, including both photo-oxidative and thermo-oxidative degradation [94]. Oxygen participates in the formation of reactive oxygen species, which are essential for the initiation and propagation of oxidative chain reactions. In aerobic environments, the presence of oxygen facilitates these oxidative processes, leading to a faster degradation of polymers. Conversely, in anaerobic conditions where oxygen is scarce, oxidative degradation is significantly slowed down. However, these conditions might enhance certain anaerobic biodegradation pathways, providing an alternative degradation mechanism [95,96].

Obviously, the degradation of polymeric materials is a complex interplay of various environmental factors. Higher temperatures, increased humidity, greater light exposure, and ample oxygen availability typically accelerate degradation processes, whereas lower temperatures, dry conditions, limited light, and reduced oxygen availability tend to inhibit them. In recent years, significant progress has been made in polymer degradation research. The impact of environmental conditions on polymer degradation processes has been extensively investigated using various methods. Table 1 presents selected studies in the field of polymer degradation in recent years, including the research methods employed, key findings, and publication dates. The goal is to reveal research trends and technological advancements, thereby providing a basis for understanding how these interactions influence predictions of polymer lifespan and for developing strategies to enhance their stability across diverse environmental contexts.

### 3.5. Other Factors

The rate and extent of polymer degradation are influenced by several additional factors, which collectively determine the stability and longevity of the material. One fundamental factor is the polymer structure. The chemical composition and molecular structure of a polymer significantly impact its susceptibility to degradation. For instance, polymers with more stable backbones, such as polytetrafluoroethylene (PTFE), exhibit greater resistance to degradation due to their robust molecular architecture [98]. On the other hand, polymers containing hydrolyzable bonds or unsaturated groups are more prone to degradation, as these structural features can be more easily attacked by environmental agents [99]. Moreover, the presence of comonomers, branching, and cross-linking within the polymer can further influence degradation behavior. For example, branching can create more amorphous regions that are susceptible to chemical attack, while cross-linking can enhance thermal and chemical resistance, thereby altering the degradation pathway [100].

Another crucial factor is the use of additives and stabilizers [101]. These compounds are often incorporated into polymers to enhance their stability and performance. Antioxidants, UV stabilizers, and heat stabilizers play a significant role in this regard by neutralizing free radicals, absorbing harmful UV radiation, and decomposing peroxides, respectively. These actions can significantly reduce the rate of degradation, thereby extending the material’s useful life [102]. However, it is also noteworthy that some additives can act as pro-degradants under certain conditions. For example, certain metal salts used as catalysts in polymer processing can inadvertently accelerate degradation by promoting oxidative reactions [103].

The physical form of the polymer is another significant determinant of its degradation behavior. The degradation rate can vary depending on whether the polymer is in the form of films, fibers, or bulk materials. Thinner materials, such as films and fibers, have a higher surface area to volume ratio, making them more susceptible to environmental factors such as light, oxygen, and moisture [104]. Consequently, these materials often degrade more quickly compared to bulk materials, which have limited surface exposure to degrading agents. Additionally, the inclusion of fillers and reinforcements within the polymer matrix can influence the degradation process. For instance, inorganic fillers can act as barriers to moisture and oxygen, thereby slowing down degradation, while organic fillers might introduce new pathways for degradation [105].

Furthermore, the processing history and physical aging of the polymer can also play significant roles in its degradation [106,107]. The conditions under which a polymer is processed, such as the temperature and pressure used during molding or extrusion, can affect its crystallinity and molecular orientation, thereby influencing its stability. Physical aging, which involves structural relaxation and densification over time, can also impact the polymer’s mechanical properties and degradation behavior.

The degradation of polymeric materials is a complex interplay of factors, including the polymer structure, the presence and type of additives and stabilizers, the physical form of the material, and its processing history and aging. This multifactorial process is driven by the interplay of physical, chemical, and biological mechanisms, influenced by various environmental conditions. A comprehensive understanding of these degradation pathways and the factors affecting them is crucial for predicting polymer stability and the environmental fate of polymeric materials. This knowledge is essential for developing effective strategies to enhance the durability and performance of polymers, advancing sustainable development, and informing environmental policies and management practices aimed at reducing plastic pollution and its associated risks.

## 4. Environmental Distribution and Pollution of Polymeric Degradation Products

Polymer materials, primarily synthetic plastics, have become integral to modern society due to their versatility, durability, and cost-effectiveness. However, their persistent nature has led to widespread environmental contamination [108]. This section examines the sources, types, and distribution of polymer materials in various environmental compartments and their associated pollution levels.

### 4.1. Distribution in Different Environmental Compartments

#### 4.1.1. Soil

**Figure 4:**
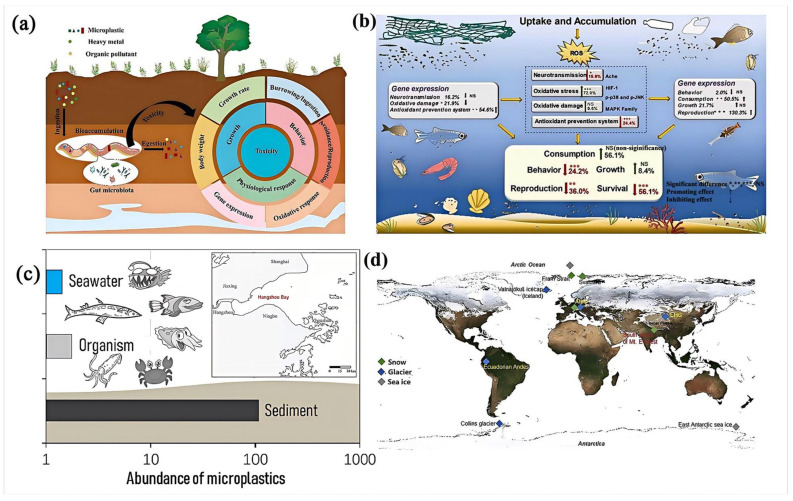
The distribution of microplastics in different environmental compartments. (**a**) Soil, reprinted with permission from [110]. (**b**) Marine environment, reprinted with permission from [111]. (**c**) Freshwater system, reprinted with permission from [114]. (**d**) Cryospheric region, reprinted with permission from [117], 2022, Elsevier.

Polymer presence in soils is primarily attributed to agricultural practices such as plastic mulching and fertilizer coatings [13], which introduce large amounts of plastic debris into the soil. Additionally, landfill leachates and littering further contribute to soil contamination. Soil amendments like sewage sludge and compost also introduce microplastics into the environment. In farmland soil, especially in soil covered with sludge or plastic, the concentration of microplastics is relatively high. Data shows that there may be hundreds to thousands of microplastics per kilogram of dry soil. The most commonly detected polymers in soil are polyethylene (PE), polypropylene (PP), polyvinyl chloride (PVC), polystyrene (PS), and polyethylene terephthalate (PET). The accumulation of these microplastics can significantly alter soil properties, affecting its porosity and water retention capabilities [109]. Moreover, the presence of microplastics can disrupt microbial activity, which is essential for nutrient cycling and maintaining soil fertility. There is growing evidence that microplastics negatively impact soil fauna, such as earthworms, which play a crucial role in soil health by aiding in decomposition and aeration. However, microplastics with higher concentrations and smaller sizes adversely affect the growth, behavior, oxidative response, gene expression, and gut microbiota of earthworms. For example, they adsorb heavy metals and HOCs and thus change earthworms’ bioavailability (Figure 4a) [110].

#### 4.1.2. Water Bodies

In freshwater systems, the major sources of polymer pollution include urban runoff, industrial discharges, agricultural runoff, and effluents from wastewater treatment plants [14]. Recreational activities and littering also contribute to the contamination of freshwater bodies. Similar to marine environments, common polymers found in freshwater systems are PET, PS, PE, and PP. The concentration range of microplastics varies from a few to tens of thousands per cubic meter. For example, thousands of microplastics per cubic meter were detected in surface water samples from the Great Lakes. The ingestion of microplastics by aquatic organisms, ranging from plankton to fish, leads to various adverse effects, such as physical blockage, reduced nutrient absorption, and the potential transfer of toxic substances. Studies have documented that nanoplastics induce oxidative stress and neurotoxicity to fish and invertebrates, and lead to negative effects on consumption, behavior, reproduction, and survival, thereby affecting the entire aquatic ecosystem (Figure 4b) [111].

In marine environments, the primary sources of polymer pollution include land-based runoff, wastewater discharge, fishing activities, and maritime transport. The degradation of larger plastic debris and the loss of fishing gear contribute significantly to the presence of microplastics in the oceans [112]. Commonly found polymers in marine environments include PET, PS, PE, and polyamide (nylon). These polymers are distributed throughout the marine environment, from surface waters to deep-sea sediments. Significant concentrations of microplastics are found in coastal regions, estuaries, and oceanic gyres. For instance, in the Great Pacific Garbage Patch [113], each square kilometer of sea area may contain over 100,000 pieces of plastic debris. Qu et al. [114] examined the abundance and characteristics of MPs in seawater, sediment, and organism samples collected from Hangzhou Bay. Figure 4c shows that microplastics are not only present in deep-sea sediments but also within marine organisms at various trophic levels, indicating a widespread environmental impact.

#### 4.1.3. Atmosphere

Polymer particles in the atmosphere originate primarily from urban dust, synthetic textile abrasion, tire wear particles, and industrial emissions [115]. Additionally, construction activities and road traffic contribute to the presence of airborne polymer particles. Common polymers found in the atmosphere include polyester, acrylic, polyamide, and polyethylene, which are often present as fibers and fragments [116]. These airborne polymers can be transported over long distances by wind and atmospheric currents, leading to their deposition in both urban and remote areas. Research has revealed the presence of microplastics in typical cryospheric regions such as the Arctic and the Alps, indicating the extensive reach and impact of atmospheric dispersion [117]. Figure 4d shows the distribution of microplastics. The widespread distribution of these particles poses potential risks to both environmental and human health, necessitating further investigation into their long-term effects.

Polymer degradation and its products vary significantly across different environmental conditions, influenced by a complex interplay of physical, chemical, and biological factors. Table 2 outlines the degradation products and features of polymers in various environmental settings. In aquatic settings, polymers degrade into small, highly persistent particles that can remain afloat for years, thereby exerting long-term impacts on aquatic life and ecosystems. Degradation in soil is predominantly influenced by microbial activity, with polymers breaking down into substances such as organic acids and carbon dioxide. However, traditional plastics may persist as microplastics, impacting the physical and chemical properties of soil. In atmospheric conditions, polymer degradation products engage in photochemical reactions, leading to the formation of photochemical smog that potentially threatens air quality and human health. Marine polymer degradation is both complex and slow, with persistent degradation products posing a grave threat to marine ecosystems and potentially impacting humans via the food chain. Each environment poses distinct challenges and impacts. Therefore, a thorough understanding of these degradation processes is essential for formulating effective environmental protection strategies, developing new biodegradable materials, and mitigating plastic pollution.

### 4.2. Pollution Levels and Trends

#### 4.2.1. Global and Regional Variations

Polymer pollution levels exhibit significant global and regional variations, with urban and industrial areas typically showing the highest concentrations. This is due to their dense populations, extensive industrial activities, and substantial waste generation. Coastal and marine environments, particularly those near urban centers and along shipping lanes, also display elevated pollution levels. Furthermore, temporal trends reveal a concerning increase in polymer pollution over the past few decades, closely linked to the exponential rise in plastic production and consumption [118]. Numerous monitoring studies have documented escalating concentrations of microplastics in both marine and freshwater environments, as well as in atmospheric deposits, highlighting the pervasive nature of this issue [119].

#### 4.2.2. Microplastics (MPs)

Microplastics (MPs) are defined as plastic particles or fragments with a diameter of less than 5 mm. They originate from two main sources: the breakdown of larger plastic debris (secondary microplastics) and direct manufacturing as small particles for specific uses (primary microplastics) [120]. Primary microplastics include microbeads from personal care products, microfibers from synthetic textiles, and industrial abrasives. On the other hand, secondary microplastics result from the fragmentation of larger plastic items due to environmental factors such as weathering, UV radiation, and mechanical abrasion.

These tiny particles have now infiltrated virtually all environmental compartments. Oceans, rivers, lakes, soils, and even the atmosphere are all contaminated with microplastics. Their presence in remote locations underscores their global distribution, carried by wind and water currents across vast distances. Alarmingly, microplastics have also been detected in drinking water and various food products [121]. Microplastics penetrate into living organisms, including humans, and accumulate in the body. Experimental studies on animals have revealed the negative effects of microplastics on health and their association with pathological changes in different organs [122]. This raises significant concerns about potential human exposure and health implications. The ubiquitous nature of microplastics and their persistence in the environment call for urgent measures to mitigate their production and release, and to understand their long-term impacts on ecosystems and human health.

### 4.3. Environmental Impact

#### 4.3.1. Ecosystem and Animal Health

The degradation of polymers in the environment is a complex and multi-stage process, influenced by various environmental and biological factors. This process not only produces intermediate products, but may also be transmitted through the food chain, ultimately affecting ecosystems and animal health. In order to more intuitively demonstrate this degradation process and its time span, Table 3 outlines the main stages of polymer exposure from the environment to its impact on animal health. This schedule helps to clearly present the connections between each stage and provides a structured framework for discussing the impact of polymer degradation products on experimental animals in the future.

The presence of polymers in the environment has profound implications for ecosystem health, particularly in marine and freshwater systems. Microplastics are ingested by a diverse array of organisms, ranging from zooplankton to fish and marine mammals. This ingestion can lead to significant physical harm, such as gastrointestinal blockages, and chemical harm from toxic additives or absorbed pollutants [123]. Furthermore, microplastics serve as vectors for invasive species and pathogens, exacerbating their impact on native ecosystems and biodiversity [124].

In terrestrial ecosystems, the effects of microplastics are equally concerning. Microplastics in soils can alter their physical structure, negatively affecting water retention and aeration [125]. This, in turn, can influence the health and function of soil microbial communities and fauna, such as earthworms, leading to disruptions in nutrient cycling and soil fertility [110]. Despite these known impacts, the long-term effects of microplastics on terrestrial ecosystems remain largely unexplored, necessitating further comprehensive research.

#### 4.3.2. Human Health Concerns

The prevalence of microplastics poses a health risk to humans. Microplastics depend on several factors, such as size, shape, and chemical composition. Studies have found microplastics in various food items, including seafood and table salt, as well as in drinking water [126]. The inhalation of airborne microplastics, present in dust and polluted air, is another significant exposure pathway. These tiny particles enter the body through various routes, with digestive tract intake, respiratory inhalation, and skin contact being the main exposure routes. Exposure to microplastics will induce cytotoxicity, cause oxidative stress, reduce immune function, and damage membranes, and then harm the human system [127].

**Figure 5:**
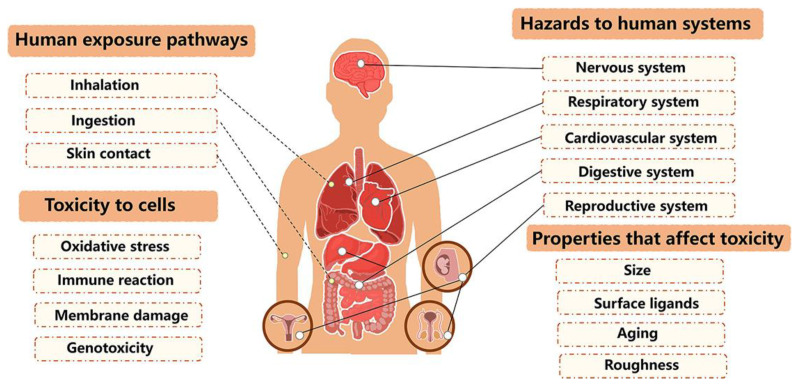
Summary of the routes, damaged systems, types of cytotoxicity, and influencing factors of human exposure to microplastics, reprinted with permission from [129], 2023, Elsevier.

Additionally, polymers can leach harmful chemicals, such as plasticizers, flame retardants, and stabilizers [80]. They also have the capacity to adsorb hazardous environmental pollutants like heavy metals and persistent organic pollutants (POPs). These substances can have severe health implications, including endocrine disruption, carcinogenic effects, and neurotoxicity [128]. Therefore, the role of polymers as carriers for these toxic substances amplifies the concern regarding human exposure and underscores the urgent need for further research into the potential health risks associated with microplastic pollution. Figure 5 summarizes the effects of microplastics on human health [129].

### 4.4. Current Status of Polymer Recycling

Polymer recycling is a complex and critical domain encompassing challenges and opportunities across technological, economic, and environmental dimensions. Currently, the global plastic recycling rate remains low; despite relatively high rates in countries like Germany (about 60%) and the United States (about 9%), the overall global rate fails to meet environmental objectives. The primary challenges include complex recycling technologies, high costs, and the diversity and pollution issues associated with plastics. Countries like Germany and Japan are leaders in recycling and reuse, employing advanced processes to convert the degradation products of polymers into industrial raw materials. For instance, waste plastics are converted into oil via pyrolysis technology, which is then used to produce new plastics [130]. Certain recalcitrant plastics are incinerated for energy recovery, converting them into heat and electricity. However, due to inadequate recycling facilities, insufficient public awareness, policy gaps, and limited economic benefits, significant amounts of polymer waste still end up in the environment, causing pollution issues. Overall, while there has been some progress in polymer recycling globally, numerous challenges remain. Enhancing technological innovation, refining policies and regulations, enhancing public awareness, and fostering international cooperation are crucial for addressing these challenges.

### 4.5. Mitigation Strategies

Addressing the environmental impacts of polymeric material degradation products necessitates a multi-faceted approach involving strategies at various levels.

#### 4.5.1. Policy and Regulation

Governments and international bodies are pivotal in implementing robust policies aimed at reducing plastic production and enhancing waste management. Bans on single-use plastics, extended producer responsibility (EPR) programs, and incentives for recycling are key measures. International agreements, such as the Basel Convention, aim to control the movement of plastic waste and ensure its environmentally sound management [131]. Moreover, regulatory measures targeting the restriction of harmful additives and the promotion of sustainable practices are essential for mitigating the adverse effects of plastic pollution.

#### 4.5.2. Public Awareness and Education

Raising public awareness through educational campaigns and community engagement is crucial in driving behavior change. Coca-Cola has launched a global environmental campaign to raise public awareness of plastic pollution and the importance of recycling, encouraging consumers to choose sustainable products. Educating the public about the environmental consequences of plastic pollution and fostering responsible consumption and waste management practices can significantly contribute to reducing plastic waste and its associated impacts [132].

#### 4.5.3. Technological Innovations

Advances in materials science are leading to the creation of biodegradable and compostable polymers, which can substantially reduce environmental persistence. Enhanced recycling technologies, such as chemical recycling and advanced sorting systems, are improving the efficiency and effectiveness of plastic waste management [133]. Furthermore, research and development efforts aimed at designing polymers with better biodegradability, recyclability, and reduced toxicity are vital for minimizing environmental pollution from plastic degradation products. For example, Ingeo PLA produced by NatureWorks is a plant-based biodegradable plastic. This material can degrade quickly under specific environmental conditions, reducing its long-term impact on the environment.

#### 4.5.4. Monitoring and Regulation

Implementing comprehensive monitoring programs to track the distribution and impact of plastic debris and degradation products in the environment is essential. These programs, in conjunction with stringent regulatory measures, help ensure the effective management and mitigation of the environmental impacts of plastic pollution [134]. Danone collaborates with research institutions to use eDNA technology for microplastic pollution monitoring in water sources to protect water resources. By continually assessing the presence and effects of plastic waste, strategies can be adapted and strengthened to address emerging challenges and protect ecosystem and human health.

The pervasive distribution and pollution of polymer materials in the environment pose significant challenges for ecosystem health and human well-being. Addressing these issues requires comprehensive monitoring, effective mitigation strategies, and continued research. Understanding the full extent of the environmental and health impacts of polymers will inform policies and practices aimed at reducing pollution and protecting the environment. Future research should focus on long-term studies, innovative materials, and sustainable practices to mitigate the adverse effects of polymer pollution.

## 5. Health Effects of Polymeric Material Degradation Products on Experimental Animals

Polymeric materials are ubiquitous in modern society, but their degradation products pose significant concerns for environmental and animal health. Understanding the diverse range of health effects induced by these degradation products is crucial for assessing their risks and developing mitigation strategies. This section comprehensively examines the various health impacts observed in experimental animal studies following exposure to plastic degradation products.

### 5.1. Organ-Specific Toxicity

Organ-specific toxicity primarily manifests in the liver and kidneys, where the accumulation of plastic degradation products can have severe implications for organ function and overall health [135]. The liver and kidneys, being critical detoxification and filtration organs, are particularly vulnerable to the adverse effects of these pollutants.

#### 5.1.1. Liver Toxicity

**Figure 6:**
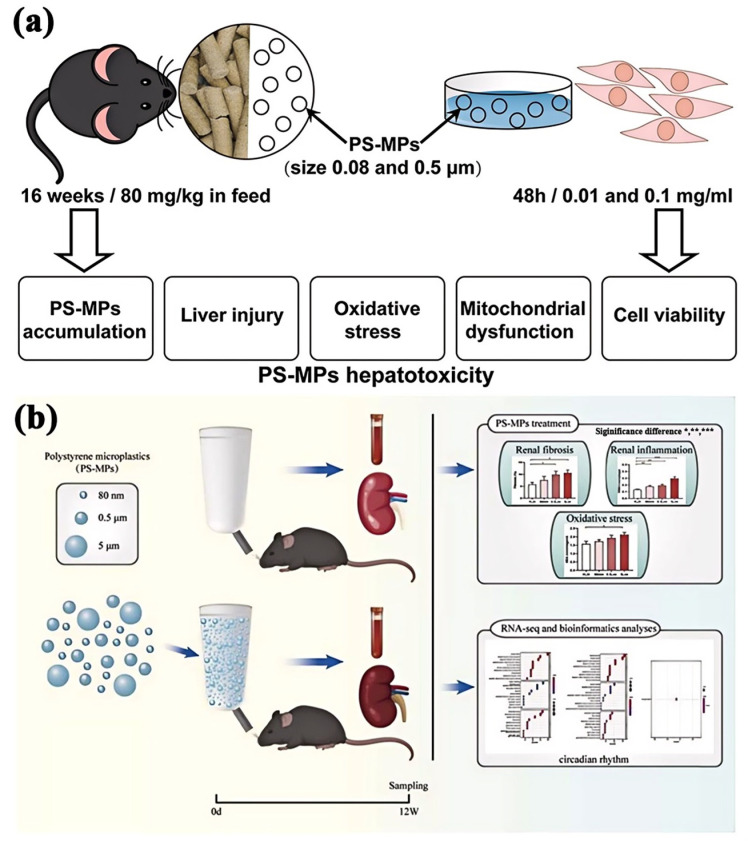
Organ-specific toxicity of PS-MPs on mice. (**a**) Liver toxicity, reprinted with permission from [139], 2024, Elsevier. (**b**) Kidney toxicity, reprinted with permission from [142], 2023, Elsevier.

Studies have consistently shown that exposure to plastic additives and degradation products leads to significant hepatotoxic effects. In rodent models, hepatotoxicity is often characterized by lipid accumulation in liver cells, which can disrupt normal liver function and lead to conditions such as fatty liver disease. Additionally, exposure to these substances triggers inflammatory responses in hepatic tissue, marked by an increase in pro-inflammatory cytokines and immune cell infiltration [136,137]. This inflammation can further impair liver function and contribute to the development of chronic liver diseases. Furthermore, plastic additives have been found to interfere with key metabolic processes in the liver, such as lipid metabolism and glucose regulation [138], potentially leading to metabolic disorders. Tao et al. [139] investigated the hepatic lipid metabolites and transcriptome of mice exposed to polystyrene microplastics (PS-MPs), revealing that PS-MPs impair glucose tolerance and induce hepatic lipid deposition in mice (Figure 6a). Su et al. [140] also demonstrated that mice exposed to Di(2-ethylhexyl) phthalate (DEHP) exhibited significant lipid accumulation, heightened inflammatory markers, and disrupted metabolic functions, and DEHP caused potential liver damage in offspring, underscoring the hepatotoxic potential of these substances.

#### 5.1.2. Kidney Toxicity

The kidneys are similarly affected by plastic pollutants, with nephrotoxicity being a significant concern. Chronic exposure to plastic degradation products has been shown to induce renal inflammation, characterized by the infiltration of immune cells and the release of inflammatory cytokines in kidney tissues [141]. This inflammatory response can lead to oxidative damage, where reactive oxygen species (ROS) generated during the inflammatory process cause cellular damage and impair kidney function. Over time, this oxidative stress can result in fibrotic changes within the renal tissue, further compromising kidney health and function. Xi et al. [142] found that MPs of different diameters caused varying degrees of damage to the mouse kidney, confirming that rodents exposed to plastic pollutants exhibited signs of renal inflammation, oxidative stress, and fibrosis, illustrating the nephrotoxic effects of these substances (Figure 6b).

### 5.2. Immunotoxicity and Inflammatory Responses

#### 5.2.1. Immune System Modulation

Plastic degradation products have been shown to modulate immune responses in experimental animal models, influencing both innate and adaptive immunity. Bishop et al. [143] showed that MP exposure suppressed innate immune responses and increased pro-inflammatory signatures. Microplastics can cause altered cytokine profiles, compromised immune cell function, and increased susceptibility to infections in specific disease settings. These immunomodulatory effects highlight the systemic impact of plastic pollution on immune homeostasis and host defense mechanisms.

#### 5.2.2. Inflammatory Responses

Chronic exposure to plastic pollutants can trigger inflammatory responses characterized by elevated levels of pro-inflammatory cytokines, reactive oxygen species (ROS), and oxidative stress markers in exposed animals. Zhang et al. [144] studied the toxicological effects of microplastics exposure on the spleen of developmental Japanese quail. They found that microplastics induced inflammatory responses and cell apoptosis in the spleen through p38 mitogen-activated protein kinases (p38 MAPK) pathway activation and tumor necrosis factor (TNF) signaling stimulation. Persistent inflammation contributes to tissue damage, organ dysfunction, and the exacerbation of underlying health conditions, underscoring the role of inflammatory pathways in mediating the adverse effects of plastic degradation products.

### 5.3. Genotoxicity and Carcinogenicity

Plastic additives and breakdown products have been implicated in inducing genotoxic effects in experimental animal models. Sarma et al. [145] revealed DNA damage, chromosomal aberrations and mutations in somatic cells exposed to plastic pollutants. These genetic alterations have implications for long-term health outcomes, including increased cancer susceptibility and heritable mutations in exposed populations.

Xiao et al. [146] found that bisphenol A and phthalate possess carcinogenic properties, contributing to the development of tumors and neoplastic lesions in animals following prolonged exposure. Carcinogenicity studies emphasize the importance of comprehensive risk assessments and regulatory measures to mitigate the carcinogenic risks associated with plastic pollution.

### 5.4. Behavioral and Neurological Effects

#### 5.4.1. Anxiety and Depression

**Figure 7:**
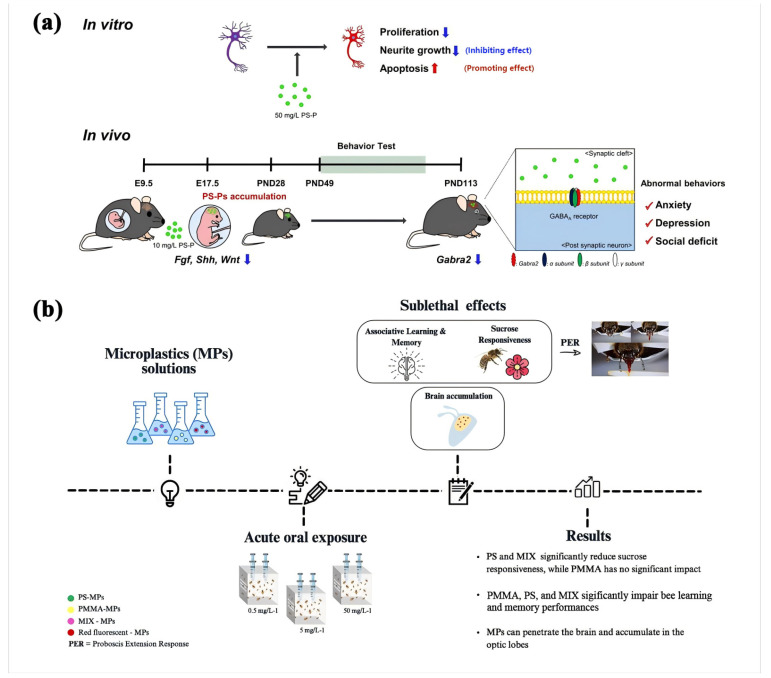
Neurotoxicity caused by animal exposure to polymeric material degradation products. (**a**) Mice exhibited depression and anxiety, reprinted with permission from [148], 2023, Elsevier. (**b**) Bees showed learning ability and memory retention impairment, reprinted with permission from [149], 2024, Elsevier.

Neurotoxicity due to abnormalities in neurons could induce neurobehavioral deficits. Ma et al. [147] indicated that chronic exposure to nanoplastics (NPs) can induce anxiety-like behaviors, depressive symptoms, and alterations in social interactions in mouse models. Similarly, Shin et al. [148] also observed an anxiety- and depression-like behavior in the offspring of PS-NP-exposed pregnant mice (Figure 7a). In addition, PS-NP exposure also altered the social behavior of mice, especially in the groups exposed to PS-COOH and PS-NH2. These behavioral changes are attributed to neurochemical imbalances, stress responses, and disruptions in neuronal circuitry associated with plastic-induced neurotoxicity.

#### 5.4.2. Cognitive Function

Pasquini et al. [149] confirmed the effects of plexiglass (PMMA) and polystyrene (PS) on cognitive function in bees via the acute oral toxicity test, manifested as decreased learning ability and impaired memory retention (Figure 7b). Neurobehavioral assessments reveal neurotoxic effects characterized by altered neurotransmitter levels, synaptic dysfunction, and behavioral deficits in exposed populations.

### 5.5. Metabolic and Endocrine Disruption

#### 5.5.1. Metabolic Disorders

Shi et al. [150] discovered that plastic pollutants are implicated in metabolic dysregulation, insulin resistance, and even diabetes by conducting a gut–liver axis experiment on mice exposed to polystyrene (PS) microplastics. Metabolic studies underscore the role of plastic-induced endocrine disruption in altering metabolic homeostasis and increasing susceptibility to metabolic disorders in exposed populations.

#### 5.5.2. Endocrine Disruption

Endocrine-disrupting effects have implications for fertility outcomes, sexual development, and hormonal balance in exposed individuals. For example, Biemann et al. [151] reveal that plastic additives such as phthalates and bisphenols can interfere with hormone signaling pathways, disrupting reproductive health, developmental processes, and metabolic regulation in animals.

### 5.6. Developmental and Reproductive Effects

#### 5.6.1. Developmental Toxicity

Exposure to plastic degradation products during critical developmental stages can disrupt embryonic development and fetal growth in experimental animal models. Chen et al. [152] reported morphological abnormalities, altered gene expression profiles, and developmental delays in offspring exposed to plastic additives during gestation. Rong et al. [153] showed polystyrene microplastics can aggravate the toxicity of environmental endocrine infections, leading to death and deformity in the offspring. These findings suggest that plastic pollutants may exert teratogenic effects, compromising embryonic viability and developmental trajectories in exposed populations.

#### 5.6.2. Fertility and Reproduction

**Figure 8:**
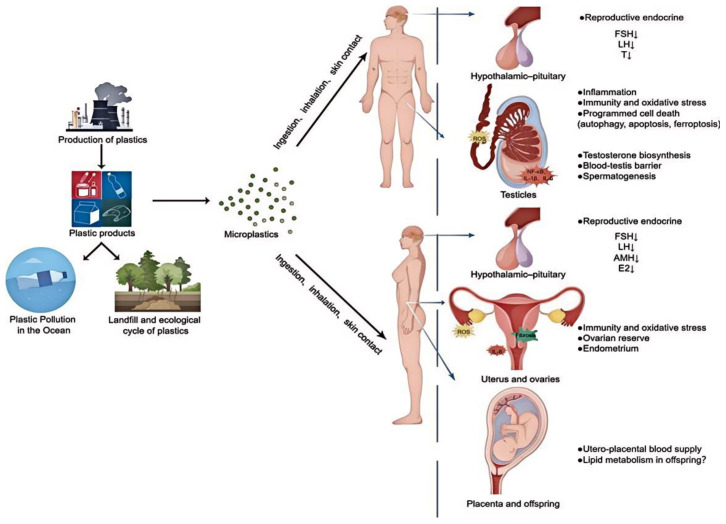
The toxicity of exposure to the microplastic environment to the reproductive system of males and females, reprinted with permission from [154], 2024, Elsevier.

Reproductive toxicity studies indicate that microplastics disrupt the neuroendocrine system, influencing sex hormone synthesis through the hypothalamic–pituitary–gonadal (HPG) axis, disturbing the reproductive health and fertility in both male and female animals (Figure 8) [154]. Microplastics interfere with the blood–testis barrier in male rodents, leading to reduced sperm quality, altered hormone levels, and impaired fertility. Jin et al. [155] showed that PS-MPs induced male reproductive dysfunctions in mice. Female reproductive health is similarly affected, such as through placental dysfunction, ovarian atrophy, endometrial hyperplasia, and fibrosis, and with evidence of disrupted estrous cycles, hormonal imbalances, and adverse effects on ovarian function following exposure to plastic degradation products. Zhang et al. [156] found PE-MPs reduced oocyte maturation, fertilization rate, and embryonic development. These findings underscore the reproductive hazards posed by plastic pollutants and emphasize the importance of assessing long-term fertility outcomes in exposed populations.

### 5.7. Long-Term Health Implications

#### 5.7.1. Cumulative Effects

Longitudinal studies in rodents and other animal models provide insights into the cumulative effects of chronic exposure to plastic pollutants on overall health and lifespan. Qiu et al. [157] demonstrated that the gene expression of the aging process is severely decreased upon exposure to nano-polystyrene. Li et al. [158] also confirmed that altered gene expression shortens the lifespan of Caenorhabditis elegans. These studies assess disease incidence, mortality rates, and age-related changes in physiological function, highlighting the enduring impact of plastic degradation products on long-term health outcomes.

#### 5.7.2. Transgenerational Effects

Emerging evidence suggests that exposure to plastic degradation products can induce epigenetic modifications and the transgenerational inheritance of adverse health effects in the offspring of exposed animals. Zaheer et al. [159] observed autism-like features in female offspring exposed to PE (10–20 mm), including repetitive and compulsive behavior. Furthermore, Teng et al. [160] showed that the exposure of the paternal zebrafish to NPs causes the clustering of NPs in the gut and brain of the offspring. Transgenerational studies underscore the intergenerational transmission of plastic-induced phenotypes and emphasize the need for multi-generational assessments in risk evaluations.

Experimental animal studies provide critical insights into the diverse health effects of plastic degradation products, encompassing acute toxicity, developmental impacts, immunotoxicity, genotoxicity, behavioral changes, and long-term health implications. These findings underscore the complex nature of plastic pollution and the urgent need for sustainable strategies to mitigate environmental and health risks associated with plastic degradation products. Continued research efforts are essential to unraveling the mechanisms of toxicity, informing regulatory decisions, and promoting the development of eco-friendly alternatives to conventional plastics.

## 6. Exposure of Experimental Animals and Impact Assessment

Experimental animals can be exposed to polymer materials through various environmental pathways, including inhalation, ingestion, and dermal contact. Inhalation involves animals breathing in airborne polymer particles or microplastics that are present in dust or suspended in the air, often originating from the breakdown of larger plastic items or industrial emissions. This can lead to the deposition of particles in the respiratory tract, causing potential irritation, inflammation, or deeper penetration into the alveoli, resulting in more severe health issues [161,162]. Ingestion is another significant pathway where animals may consume polymer particles or microplastics through contaminated food or water. This is particularly relevant in studies simulating environmental exposure where animals are provided with intentionally contaminated food or water. Zhao et al. [163] explored whether consuming microplastics promotes preclinical cardiovascular disease (CVD) by feeding mice with water containing polystyrene. Behavioral ingestion, such as rodents nibbling on their surroundings, also contributes to this pathway. Ingested polymers can cause physical blockages, an irritation of the gut lining, and potential uptake into systemic circulation depending on particle size and polymer composition [164]. Although less common, dermal contact occurs when animals come into direct contact with polymer materials present on surfaces or in bedding. This can lead to local irritation or the absorption of smaller particles through the skin, with the potential for transdermal absorption depending on the size, chemical properties, and duration of exposure [165]. Compromised skin, due to injury or illness, is known to be more permeable and is a possible route for unintended microparticle absorption stemming from environmental exposure [166].

Studies on the exposure of experimental animals to polymers are categorized into acute and chronic exposure assessments. Acute exposure studies involve short-term, high-dose exposures to identify immediate toxicological effects, such as immune dysregulation, gastrointestinal upset, or immediate behavioral changes. Cappello et al. [167] confirmed that during the short-term (72 h) exposure period, the intracellular ROS levels in mussels significantly increased, causing dynamic disturbances in energy metabolism in the digestive glands of mussels attacked by MPs. Gaspar et al. [168] found that short-term exposure in mouse liver and brain tissue resulted in the accumulation of MPs, changes in cognitive behavior, and alterations in immune markers in liver and brain tissue. These studies often include the detailed monitoring of clinical signs and histopathological examinations post-exposure to reveal tissue-specific damage. Chronic exposure studies, on the other hand, are designed to mimic real-world scenarios where animals are exposed to lower doses of polymers over extended periods. Bao et al. [169] reported that long-term exposure to PLA MPs in tilapia can lead to more severe dysbiosis of the gut microbiota, resulting in damage to intestinal tissue. Song et al. [170] also confirmed that high-dose MPs promote oxidative stress and an inflammatory response in cardiomyocytes that damages myocardial tissue. These studies provide insight into cumulative effects and potential long-term health impacts. Researchers track various health parameters over time, including weight changes, organ function, reproductive health, and long-term behavioral changes. Life-long exposure assessments are also conducted to evaluate lifespan and disease onset.

The health impact of polymer exposure on experimental animals is assessed through various biological and physiological parameters. For the respiratory system, a histopathological examination of tissue samples from the respiratory tract is conducted to identify signs of inflammation, fibrosis, and other pathological changes. Inflammatory markers in the blood or lung tissue, along with pulmonary function tests, help determine the impact on respiratory efficiency [171]. In the gastrointestinal system, histological analysis is performed to detect physical damage, inflammation, and changes in tissue structure. Luo et al. [172] reported that PS MPs exacerbated intestinal inflammation induced by DSS in colitis, reduced mucus secretion, and increased intestinal permeability. Studies also assess changes in gut microbiota composition and function, as well as the efficiency of nutrient absorption [173,174]. Reproductive and developmental effects are evaluated through fertility studies, monitoring embryonic development, and assessing offspring viability to determine generational impacts of polymer exposure.

Understanding the molecular mechanisms underlying polymer-induced toxicity is essential for comprehensive impact assessment. Oxidative stress is measured by assessing reactive oxygen species (ROS) levels, antioxidant enzyme activities, and lipid peroxidation products [20]. Inflammatory responses are evaluated by measuring cytokine levels, analyzing immune cell activation, and examining tissues for histological signs of inflammation [136]. Genotoxicity and carcinogenicity are assessed through DNA damage assays, mutagenesis studies, and long-term carcinogenicity studies [144]. Endocrine disruption is evaluated by measuring hormone levels, conducting receptor binding studies, and monitoring reproductive health indicators [151].

The findings from experimental animal studies are crucial for risk assessment and regulatory decision-making. Establishing dose–response relationships helps to determine threshold levels for safe exposure and identify no-observed-adverse-effect levels (NOAEL) and lowest-observed-adverse-effect levels (LOAEL) [175]. Quantitative risk assessments combine exposure data with dose–response relationships to characterize the risk to health. Data from animal studies inform the development of safety guidelines and regulatory standards for permissible levels of polymer materials in the environment. These guidelines ensure the protection of public health by establishing health-based criteria for the presence of polymers in food, water, air, and consumer products. Additionally, research outcomes contribute to policy development aimed at reducing environmental polymer pollution through improved waste management, recycling, and the development of alternative materials. Public health initiatives also play a role in raising awareness about the potential health risks of polymer exposure and promoting safer practices in industries and communities.

The toxicological impacts of degradation products from various polymers on experimental animal models are a significant area of current research. These studies demonstrate how different chemicals and particles, resulting from the environmental degradation of various polymers, affect both organismal health and ecosystems (as detailed in Table 4). Research into the toxicity of these degradation products in experimental animal models aids scientists in understanding the potential threats they pose to ecosystems and human health. This, in turn, informs the development of safer standards and protection policies for material management and environmental regulation.

## 7. Comprehensive Impact of Polymeric Material Degradation on Environmental Health

Polymeric materials, ubiquitous in modern society, pose significant environmental challenges due to their persistence and widespread distribution. Understanding the comprehensive impact of plastic degradation on environmental health involves examining ecological disruptions, chemical contamination, and the long-term sustainability implications.

### 7.1. Ecological Disruptions

The presence of degradation products from polymeric materials in the environment can have significant ecological impacts. Microplastics, when ingested by marine organisms such as fish, seabirds, and marine mammals, can cause physical harm and blockages in digestive systems, and reduce feeding efficiency. The ingestion of microplastics can also lead to the bioaccumulation and biomagnification of toxic chemicals adsorbed onto their surfaces, such as persistent organic pollutants (POPs) and heavy metals [128]. These contaminants can accumulate in tissues over time, posing risks to individual organisms and potentially affecting entire ecosystems. In addition, chemical breakdown products released during the degradation of plastics, such as bisphenol A (BPA) from polycarbonate or phthalates from PVC, are known for their endocrine-disrupting properties [151]. These compounds can interfere with hormone systems in animals and humans, affecting reproductive health, development, and immune function. Additionally, some degradation products, such as polycyclic aromatic hydrocarbons (PAHs), are carcinogenic and mutagenic, posing direct health risks to wildlife and ecosystems [176].

#### 7.1.1. Habitat Alterations

Polymeric materials, especially microplastics, are prevalent in terrestrial and aquatic environments. Their accumulation alters natural habitats by affecting soil structure, water retention capacity, and nutrient cycling processes. In terrestrial ecosystems, plastic debris can smother vegetation and disrupt soil microbiota, leading to reduced biodiversity and impaired ecosystem services [177]. In aquatic environments, microplastics accumulate on water surfaces and ocean floors, altering light penetration and disrupting marine habitats. Floating plastic debris can also create artificial substrates that alter coastal and marine ecosystems, affecting species distribution and ecosystem functioning [178].

#### 7.1.2. Biodiversity Loss

Plastic pollution poses a direct threat to biodiversity through entanglement, ingestion, and habitat alteration. Marine organisms, from plankton to marine mammals, ingest microplastics, leading to physical harm, reduced feeding efficiency, and reproductive impairment [179,180]. These impacts cascade through marine food webs, affecting predator–prey dynamics and biodiversity at ecosystem scales. In terrestrial ecosystems, plastic debris poses similar risks to wildlife. Animals may mistake plastic fragments for food, leading to gastrointestinal blockages, malnutrition, and population declines. Plastic pollution also indirectly affects biodiversity by introducing invasive species transported on floating debris, disrupting native species interactions and ecosystem stability [181].

### 7.2. Chemical Contamination

#### 7.2.1. Leaching of Additives

Plastic degradation releases a myriad of chemical additives into the environment, including plasticizers, flame retardants, and stabilizers. These additives can leach from plastic particles, especially microplastics, under environmental conditions, leading to the widespread contamination of soil, water, and air. In aquatic environments, leached chemicals accumulate in sediments and water columns, posing risks to aquatic organisms. Plastic additives such as phthalates and bisphenols have been linked to endocrine disruption, reproductive abnormalities, and developmental impairments in aquatic invertebrates [182].

#### 7.2.2. Toxicological Impacts

The toxicological impacts of plastic-derived chemicals extend beyond aquatic organisms to terrestrial wildlife and humans. Wildlife species exposed to contaminated environments may exhibit physiological changes, including altered hormone levels, immune suppression, and increased susceptibility to diseases. Endocrine-disrupting chemicals found in plastic, such as phthalates, can disrupt reproductive health, leading to reduced fertility and developmental abnormalities in exposed populations [183].

#### 7.2.3. Persistent Organic Pollutants

Microplastics have a high affinity for persistent organic pollutants (POPs) such as polychlorinated biphenyls (PCBs) and pesticides. These hydrophobic compounds adsorb onto plastic surfaces in aquatic environments, concentrating over time. As microplastics are ingested by marine organisms, POPs biomagnify through marine food chains, posing risks to higher trophic levels, including marine mammals and humans consuming seafood [184].

### 7.3. Long-Term Sustainability Challenges

The durability of polymeric materials complicates waste management efforts, leading to accumulation in landfills, oceans, and remote environments. Inadequate waste disposal practices and a lack of recycling infrastructure contribute to the persistence of plastic pollution, exacerbating environmental contamination and health risks. For example, although biodegradable plastics are designed as alternatives to reduce environmental burden, they can only be effectively degraded under specific industrial composting conditions. The slow degradation rate in ordinary environments, such as landfills or natural environments, can also lead to the accumulation of microplastics and degradation products [185].

Effective waste management strategies, including recycling programs, extended producer responsibility (EPR), and plastic bans, are crucial for reducing plastic waste generation and promoting circular economy practices. These initiatives aim to minimize environmental impacts and conserve resources through sustainable plastic use and disposal practices.

Addressing plastic pollution requires comprehensive policy interventions and regulatory frameworks at local, national, and international levels. Policy measures include bans on single-use plastics, regulations on plastic manufacturing and disposal practices, and incentives for sustainable alternatives. The effective implementation of policies relies on collaboration among governments, industries, and civil society to promote innovation, research, and public awareness. By adopting evidence-based approaches and integrating stakeholder perspectives, policymakers can advance sustainable plastic management strategies and mitigate the environmental and health impacts of plastic degradation.

The comprehensive impact of polymeric material degradation on environmental health underscores the urgency of addressing plastic pollution through integrated strategies. By mitigating ecological disruptions, reducing chemical contamination, and promoting sustainable practices, societies can mitigate the adverse effects of plastic pollution on ecosystems, biodiversity, and human health. Continued research, policy innovation, and public engagement are essential for achieving sustainable plastic use and preserving environmental quality for future generations.

### 7.4. Major Progress in Polymer Removal

In recent years, significant progress has been made in removing polymers from the environment. New technologies and innovative methods are accelerating the elimination of plastic pollution. Firstly, physical removal technology is currently the most widely used polymer removal method, especially in marine, river, and terrestrial environments where it performs outstandingly. A large number of beach and river cleaning activities have been organized globally, using manual or mechanical equipment to clean up polymer waste on a large scale. For example, Boyan Slat’s ocean cleanup project has expanded to rivers, using directional devices to block and collect plastic debris in rivers, reducing ocean pollution from the source. Next is the method of biodegradation. Using microorganisms or enzyme reactions to decompose polymers is an environmentally friendly and sustainable removal strategy. Research has shown that certain bacteria and fungi are capable of decomposing specific types of polymers, and microorganisms can break down plastics into water, carbon dioxide, and other harmless byproducts through natural metabolic processes. The third is chemical treatment technology, which has made significant progress in laboratory and industrial applications. Through catalysts (such as metal oxides or organic catalysts) and heat treatment, plastics can be decomposed into monomers or fuels. These methods can convert polymer waste into useful chemical raw materials or energy, reducing the accumulation of plastics in the environment. Significant progress has been made globally in removing polymers from the environment, covering various technologies from physical cleaning to biodegradation and then to chemical treatment. In the future, with further technological development and policy support, it is expected that polymer pollution in the environment will be more effectively controlled and eliminated.

## 8. Summary and Outlook

In summary, the impact of polymeric material degradation on environmental health is profound and multifaceted. Plastic pollution disrupts ecosystems, threatens biodiversity, and introduces harmful chemicals into the environment. From terrestrial habitats to marine ecosystems, plastic debris alters natural landscapes and poses risks to wildlife through ingestion and entanglement. Chemical additives and persistent organic pollutants associated with plastics further exacerbate environmental contamination, impacting aquatic life and potentially entering the food chain. These challenges highlight the urgent need for coordinated global efforts to mitigate plastic pollution, enhance waste management practices, and promote sustainable alternatives.

Looking forward, addressing plastic pollution requires a holistic approach encompassing research innovation, policy development, and public engagement. Future research should prioritize understanding the full extent of microplastic contamination, including its sources, distribution, and ecological impacts across diverse ecosystems. Innovations in materials science offer promising avenues for developing biodegradable plastics, eco-friendly packaging materials, and sustainable manufacturing practices that reduce reliance on conventional plastics. Policy frameworks must strengthen regulations on plastic production, disposal, and recycling, while promoting international cooperation to tackle transboundary plastic pollution challenges effectively. Moreover, public awareness campaigns and educational initiatives are crucial in empowering individuals and communities to adopt sustainable behaviors and advocate for environmental stewardship. By integrating these strategies, we can strive towards a cleaner, healthier planet where plastic pollution is minimized, and environmental integrity is preserved for future generations.

## Figures and Tables

**Table 1:** Summary of the latest research on polymer degradation.

Publication Date	Degradation Mechanism	Research Methods	Key Findings	Ref.
January 2023	Thermo-oxidative degradation	Thermogravimetric analysis	Found that high temperature can alter the thermal degradation performance of PVC.	[79]
June 2023	Photocatalytic degradation	Thermogravimetric analysis, visible light irradiation test	The effectiveness of bio-inspired C,N-TiO_2_/SiO_2_ photocatalysts in the degradation of PET microplastics was discovered.	[97]
January 2024	Hydrolytic degradation	Hydrolysis test, platelet adhesion test	Demonstrated the effects of an amide linker on the hydration and hydrolytic properties of APCs.	[82]
August 2022	Enzymatic degradation	Establishment of enzyme reaction system, enzyme catalyzed degradation	Described the synergistic effect of keratinase hydrolysis carboxylesterase in PET degradation.	[86]
May 2023	Microbial degradation	Microbial cultivation screening, biodegradation test	Explored the effect of the structure–activity relationship of microorganisms on the biodegradability of polymers.	[88]

**Table 2:** Degradation products of the polymers under different environmental conditions.

Environmental Conditions	Main Degradation Pathways	Degradation Products	Persistence
Water	Hydrolytic degradation, UV degradation	Microplastics, nanoplastics, low molecular weight compounds	Medium to high persistence, possibly several years or even longer
Soil	Microbial degradation, chemical degradation	Organic acids, CO_2_, methane, microplastics	Low to moderate persistence
Air	Thermal degradation, photo-oxidative degradation	CO_2_, low molecular weight volatile organic compounds	Low to moderate persistence
Sea	Hydrolytic degradation, UV degradation	Microplastics, nanoplastics, low molecular weight compounds	High persistence, ranging from years to decades

**Table 3:** Degradation process of polymers from environmental exposure to animal health impacts.

Time Period	Process Description	Impact Analysis
Day 0	Environmental exposure, polymer release into the environment (such as landfill and industrial emissions).	Polymers begin to be exposed to nature and are affected by factors such as ultraviolet radiation, temperature, and humidity.
Day 1–30	Initial degradation, physical and chemical degradation, begins.	Molecular weight decreases and surface degradation occurs.
Day 30–90	Further degradation of polymers produces intermediate products (small molecule compounds or microplastic particles).	Intermediate products may enter the environment and affect ecosystems.
Day 90–180	Biodegradable polymer products (microorganisms or enzymes) generate smaller molecules or harmless substances.	Degradation is deeper under biological action, and some substances may be toxic.
Day 180–Year 1	Partial incomplete degradation products accumulate in the environment and may be transferred upwards through the food chain.	The long-term accumulation of products, which may enter water sources, the soil, and the atmosphere, affecting the health of animals and plants.
Year 1–5	Degradation products or microplastics are ingested by animals through the food chain, affecting their physiological functions.	Potential toxic effects, including visceral damage, metabolic disorders, etc.

**Table 4:** Toxicological effects of polymer degradation products and their impact on experimental animals.

Polymer Type	Degradation Products	Exposure Pathway	Observed Effects	Relevance to Human Health
Polylactic acid (PLA)	CO_2_, H_2_O	Oral administration, injection, skin contact	Changes in weight and blood biochemical indicators	Less restricted and generally considered a relatively safe degradation product.
Polybutylene succinate (PBS)	Succinic acid, butanediol	Oral administration, injection	Liver and kidney damage, blood toxicity	Further research is needed on the potential impact of long-term exposure on human health.
Polyethylene (PE)	Microplastic particles	Inhalation, skin contact	Allergic reactions, respiratory system issues	May have a significant impact on individuals with allergies.
Polypropylene (PP)	Succinic acid, butanediol	Inhalation, injection	Immune system response, nervous system damage	May lead to immune and neurological health issues.

## Data Availability

The original contributions presented in the study are included in the article, further inquiries can be directed to the corresponding author.
